# Carboxymethyl Cellulose (CMC) Based Electrospun Composite Nanofiber Mats for Food Packaging

**DOI:** 10.3390/polym13020302

**Published:** 2021-01-19

**Authors:** Motahira Hashmi, Sana Ullah, Azeem Ullah, Yusuke Saito, Md. Kaiser Haider, Xinyu Bie, Kosei Wada, Ick Soo Kim

**Affiliations:** Nano Fusion Technology Research Group, Shinshu University Ueda Campus, Nagano 386-8567, Japan; motahirashah31@gmail.com (M.H.); sanamalik269@gmail.com (S.U.); 08tex101@gmail.com (A.U.); 19fs311d@shinshu-u.ac.jp (Y.S.); kaisershakil@yahoo.com (M.K.H.); 19fs325d@shinshu-u.ac.jp (X.B.); 20fs326h@shinshu-u.ac.jp (K.W.)

**Keywords:** electrospinning, food packaging, carboxymethyl cellulose, air permeability, sustainable

## Abstract

Cellulose is one of the most abundantly available natural polymers. Carboxymethyl cellulose (CMC) belongs to the cellulose family and has different degrees of substitution. Current research comprises the fabrication and characterization of CMC nanofibers using polyvinyl alcohol (PVA) and polyvinylpyrrolidone (PVP) as capping agents and carriers for sustainable food packaging applications. Recently authors successfully fabricated smooth and uniform nanofibers of stated polymers and optimized the ratios of three polymers for continuous production. However, in this research, it was further characterized for mechanical properties, surface properties, structural properties, air permeability, and chemical properties to confirm the suitability and scope of tri-component nanofibrous mats in food packaging applications. Different fruits and vegetables were packed in a plastic container and closed by nanofiber mats and by a plastic lid. All samples were observed after a specific period of time (fruits were kept for 40 days while vegetables were kept for 10 days in the controlled environment). It was observed in the results that fruits and vegetables closed by nanofiber based webs exhibited better freshness and lower accumulation of moisture as compared to that of containers with plastic lids. From the results of performed tests, it was observed that nanofiber mats possess enough mechanical, structural, and morphological properties to be used as food packaging.

## 1. Introduction

Sustainability is one of the key considerations while developing materials, methods, or systems. Materials from sustainable sources are always preferred over unsustainable materials. Natural polymers have a vast range of applications in every area of science [1,2,3,4,5,6]. However, the main problem associated with natural polymers is difficulties in processing to make them usable as useful products. Polylactic acid (PLA), chitosan [7,8], cellulose [2,3,9], zein [9], silk, and other polymers are some of the most used polymers in the field of biomedical engineering, environmental engineering, food packaging [10], tissue engineering, and other applications. Natural polymers are usually modified or blended with synthetic polymers to increase application areas due to higher productivity and smoother processing of synthetic polymers [1,7,8,11,12,13,14,15]. Nanofibers fabricated by either production method have multiple application areas, especially in life science (tissue engineering, biomedical engineering, including implants, wound dressing, drug delivery, etc.), food packaging, energy storage, and filters for air [16,17] and water purification, and membranes. Electrospun nanofibers have also played a key role in the fight against COVID-19 [18,19,20]. Electrospinning is a facile and easy approach to produce fine fibers in the nanometers range [21,22]. That is why electrospun nanofibers have a wider application area as compared to that of melt spinning or others [23,24,25,26,27,28,29]. When it comes to sustainable development goals (SDGs), hygienic food availability becomes an essential consideration. Different food packaging based on natural or synthetic polymers is available in markets to preserve food for longer shelf life [30,31,32]. However, nanofiber based food packaging is not common due to the cost, production, and availability of nanofibrous mats [33,34,35]. Depending on the type of food, preservation conditions also vary. It is essential to develop functional polymers based food packaging, which can sustain in varying conditions (temperature, humidity, bio-organisms, etc.) [36,37,38,39,40]. CMC got the attention of researchers due to its unique characteristics and increasing demands in various applications in different areas like the food industry including food packaging, tissue engineering, drug delivery, printing, cosmetics, and dying. CMC, due to its good water interaction and water holding capacity (WHC), has also applications as absorbent. Mostly materials show better water holding capacity at high temperatures but it is the specialty of CMC that it shows excellent WHC even at low temperatures. CMC possesses excellent water absorption properties both in film and fiber form but the rate of absorption is lower in the fiber form than film. In CMC fiber absorption of 2000% of water from its initial mass has been reported while it was 6000% in case of CMC film. The presence of hydroxyl groups imparts moisture absorbing properties in CMC. The presence of -OH functionalities work as the binder (binding forces) for holding excess amount of water because of hydrogen bonding [6,11].

CMC is easily soluble in water and a hydrophilic polymer. CMC is soluble in cold water and does not form a gel. There are unsubstituted sites present on the backbone of CMC, which lowers the solubility of CMC in cold water. In cold water the unsubstituted sites of CMC do not work properly but as the water temperature goes up gel formation rate increases because of unsubstituted cellulose sites along the backbone chain as temporary cross-linkers between the chains. Viscosity of CMC and gel formation rate is directly proportional to each other. Increase in viscosity makes the CMC difficult to electrospin. PVA is well renowned polymer due to its easy spinnability, biocompatibility, and good thermo mechanical properties [41,42]. Hydroxyl groups are present on PVA, which cause inter and intramolecular hydrogen bonding. CMC and PVA together possess the strong interaction due to the hydrogen bonding. Both polymers are known for their solubility in water, which leads them towards the homogeneous solution [8,12,43]. PVP is famous due to its hydrophilicity. It also have good conductivity, biocompatibility, excellent film-forming properties, non-toxicity, biodegradability, and low surface tension [7,44]. PVP makes strong interaction with both CMC and PVA. The presence of carbonyl groups on PVP chain and hydroxyl groups on PVA chain leads them towards the strong interchain hydrogen bonding. Together CMC, PVP, and PVA possess excellent biocompatibility, biodegradability, spinnability, low surface tension, and good conductivity, which make it the most suitable material for active food packaging. There are different types of available food packaging, which include preharvest, post-harvest, active packaging, smart and intelligent packaging, and so on. All of the stated food packaging have a significant role in preserving food and maintaining freshness over a longer period of times. Our recent research targets post-harvest packaging application of electrospun nanofibers based on CMC, which is derived from a sustainable source (cellulose). Nanofibrous food packaging has a number of advantages over film based packaging due to unique morphology and a structure of nanofibrous membranes (in the results section it can be viewed by how different it is). In our previous research [45] the loading capacity of CMC was optimized for smooth nanofiber production without beads formation, however, further crosslinking and practical applicability was not examined. Current research is focused on developing food packaging that is superior in terms of structural and mechanical properties and fulfilling one of the goals of SDGs, which is sustainability. The primary source of CMC is cellulose, which is a natural and sustainable polymer. Consuming cellulose in the form of nanofibers is one of the suitable ways to boost the usability of cellulose. CMC nanofibers can potentially be used as food packaging to secure food (mostly fruits and vegetables) for a longer time.

## 2. Materials and Methods

Polyvinylpyrrolidone (PVP) (average molecular weight of 40,000 g mol^−1^), polyvinyl alcohol (PVA) (average molecular of 85,000–124,000 g mol^−1^, hydrolyzation degree of 87–89%), and sodium carboxymethyl cellulose (Na-CMC) (average molecular weight of 250,000 g mol^−1^ with substitution degree of 1.2) were procured from Sigma-Aldrich (Lois, USA). Polymers were not further modified physically or chemically before using.

PVA concentration in spinning solution was fixed at 6% *w/w* while weight percentage of PVP was standardized at 12% *w/w*. CMC was added in 3 different concentrations i.e., 16.6%, 33.4%, and 50% by weight of PVA (as PVA was selected as the base material/carrier; mentioned as ratios of PVA:PVP:CMC). Added in each solution 30 min before electrospinning was 2% glutaraldehyde (25% dilution) (Table 1 shows sample details). Electrospinning conditions were set to as follows; syringe 20 mL, stainless steel needle of 18 gauge, commercially available syringe pump (SPS series, AS One), flow rate 0.8 mL/h, tip to collector distance was kept at 14 cm, and voltage of 20 kV was applied. After electrospinning all samples were crosslinked by the HCl fume method as per our previous research [46].

## 3. Characterizations

Possibility of any chemical interactions among functional sides of PVA, PVP, and CMC, PVP was examined using Fourier transform infrared spectroscopy (FTIR) with an attenuated total reflection (ATR) prestige-21 (Shimadzu, Japan). FTIR-ATR spectra were taken from wave number ranging from 600 to 4000 cm^−1^. Prepared nanofibers’ morphology was examined using the scanning electron microscope (SEM) (JSM-5300, JEOL Ltd., Osaka, Japan). Acceleration energy was kept at 10 kV for all samples. Diameters of nanofibers were calculated by Image J. software and taking average of 50 random readings from different nanofibers from each image. The degree of hydrophilicity of prepared nanofibrous mats was confirmed by measuring the water contact angle (WCA) of each nanofibrous sample. WCA was measured by using the contact angle analyzer (Digidrop, GBX, Whitestone way, Croydon, UK). Degradation behavior of nanofibers under high temperatures was analyzed using the thermogravimetric analyzer (Thermo-plus TG 8120, Rigaku Corporation, Osaka, Japan). The thermal degradation test was carried out in ambient atmosphere (in ambient air) and static mode while the incremental rate of heating was 10 °C/min in temperature range of 25–500 °C. Sample weight for all specimen was 10 mg each. Crystalline structure of CMC based composite nanofibers was observed by the X-ray diffraction (XRD) spectra of each sample. XRD spectra were recorded at room temperature (25 ± 3 °C) using Rotaflex RT300 mA, Rigaku, Osaka, Japan, an angle (2θ) ranging from 5 ≤ 2θ ≤ 70°. Mechanical properties of prepared composite nanofibers were assessed by the universal testing machine (UTM), Tensilon RTC 250A; A&D Company Ltd., Osaka, Japan. ISO 13634 testing standard was followed to prepare and characterize all specimens (note that *n* = 5 for each type of nanofibers), crosshead speed was 5 mm/min and the test was performed at room temperature. Air permeability of CMC based electrospun nanofibers was performed using Air Permeability Tester Kato Tech Co., Ltd., Osaka, Japan. Average resistance (R) was calculated by measuring the AP of 5 specimens for each type of nanofibers. Suitability of electrospun nanofibers for food packaging applications was assessed by comparing nanofibers and plastic packaging for different fruits and vegetables in controlled environment (relative humidity 75% and temperature 2 °C (275 K)).

## 4. Results and Discussions

### 4.1. Fourier Transform Infrared Spectroscopy (FTIR)

**Figure 1:**
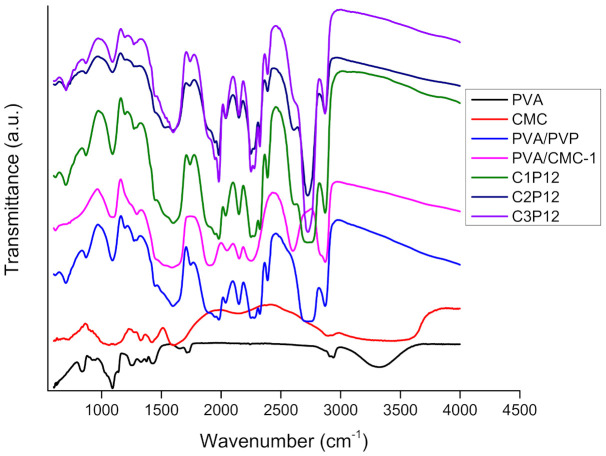
FTIR-ATR spectra of PVA, CMC, PVA/PVP, and tricomponent PVA/PVP/CMC electrospun nanofibers (all spectra are shifted vertically for clarity).

Abundant availability of hydroxyl groups in the main chains of PVA, PVP, and CMC makes these polymers highly hydrophilic. In our recent research, FTIR spectra of uncrosslinked CMC based nanofibers have been explained [45]. However after crosslinking all nanofibrous mats were become hydrophobic because of binding of hydroxyl groups in the result of reaction supported by glutaraldehyde and HCl. FTIR-ATR spectra in Figure 1 represents interactions among PVA, CMC, and PVP. FTIR spectra of pure CMC was taken as CMC was in the powder form (because nanofibers of pure CMC were not formed), so an –OH peak was observed in pure CMC spectrum. Even after crosslinking by the HCl fumes method, there was still a peak of -OH bond, which is the representative peak of the hydroxyl group, and was present in the ATR spectrum of pure PVA as it can be seen in Figure 1, at a wavenumber range of 3000–3550 cm^−1^ a broader peak, which was associated with the hydroxyl group in PVA and CMC main chains, was observed in the ATR spectra. However, the stated peak was becoming more flatter in the case of CMC based composite nanofibers. It was concluded in the results of WCA that CMC based composite nanofibrous mats presented a bigger contact angle (˃90°). Characteristic peaks of PVA were observed at 3200–3450 cm^−1^, which indicates the presence of the hydroxyl group (–OH stretching) in PVA chains (however limited due to crosslinking but still available). No hydroxyl peak was found in the case of a composite nanofiber, which indicates completely restricted –OH functionality in composite nanofibers. In the PVA spectrum, the symmetric and asymmetric peak (–CH_2_–) was observed at 2900 cm^−1^ [46], while PVP polymer exhibited its characteristic peak of carboxylic groups’ (C=O) stretching vibration in the pyrrolidone structure at 1650 cm^−1^, and peaks from 2850 to 2980 cm^−1^ were associated with CH stretching. Peaks at 1430 cm^−1^ and 1370 cm^−1^ were associated with the CH deformation band (difference in peaks in red and black colors in Figure 1). Bending vibrations of the C–N band in pyrrolidone functionality were associated to the peaks wavenumber at 1279 cm^−1^. The presence of any amine groups was not found in the PVP spectrum as PVP did not show any significance peak at 3400–3500 cm^−1^ [47]. In conclusion of the chemical characterization, it was concluded that hydroxyl groups in main chains of stated polymers were bound because of crosslinking with the possibility of hydrogen bonding as well (however, no significance peak shift was observed except flattening of the hydroxyl peak of composite nanofibers) because significant improvement was observed in tensile and water contact angles.

### 4.2. Morphological Properties

**Figure 2:**
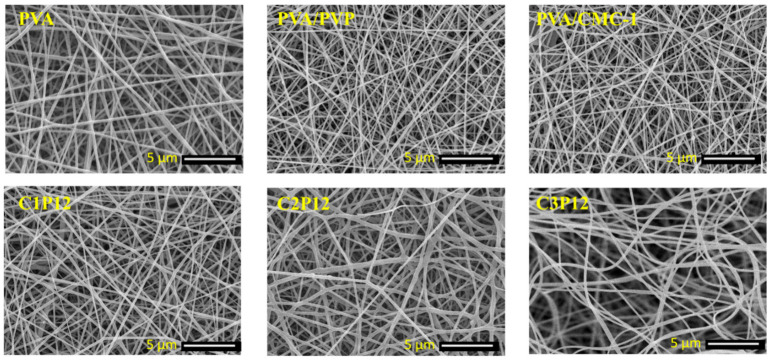
Scanning electron microscopic images of PVA, PVA/PVP, and tricomponent PVA/PVP/CMC nanofibers (each SEM image has a navigation bar that shows that the scale bar for all specimen was 5000 nm and acceleration energy was 10 kV).

Surface structure and diameter of nanofibers were examined by scanning electron microscope (SEM). It is already stated that it is very difficult to electrospin CMC due to its high viscosity and poor conductivity. However, PVA can be easily electrospun even at a different concentration but a concentration from 6% to 10% is the most appropriate to get smooth nanofibers. PVP possesses low viscosity at lower concentrations, which makes it difficult to electrospin. PVP helps to decrease the viscosity of CMC and imparts conductivity in it. CMC and PVP together give ideal viscosity and conductivity, which show results in the form of good nanofibers. In our recent research the concentration of PVP and CMC was optimized to obtain uniform nanofibers. SEM images of CMC based electrospun nanofibrous mats and neat PVA nanofibers are shown in Figure 2. It was observed that PVA/CMC-1 (PVA:CMC = 6:1) nanofibers were beads-free and smooth but as the concentration of CMC increased to 6:2 (*w/w*) not a single nanofiber was observed. Nanofibers of PVA/PVP composite were also smooth as it can be shown in Figure 2. C1P12, C2P12, and C3P12 are the samples with the addition of PVP in spinning solution, which impart conductivity and lower the viscosity of solution. Smooth and fine nanofibers can be observed from Figure 2. In the final remarks of morphological characterization, it can be stated that maximum and optimum loading capacity for CMC was up to 50% (*w/w*) of its carrier PVA. So, the authors recommended that PVA:PVP:CMC = 6:12:3 concentration is most suitable for smoother and continuous nanofibers production without beads formation.

#### Diameter Distribution of Nanofibers

**Figure 3:**
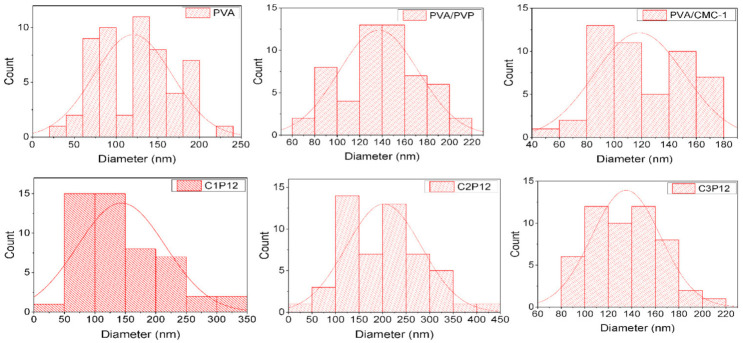
Diameter distribution of PVA, PVA/PVP, and tricomponent PVA/PVP/CMC nanofibers (the X-axis shows the diameter in nm, while the Y-axis shows the number of fibers in that diameter range).

Measuring diameters of nanofibers is one of the important tools to assess uniformity of mats. Diameters of the CMC based composite, PVA/PVP, and PVA nanofibers were calculated by taking 50 readings of random nanofibers using Image J. software. Figure 3 (histograms) shows the distribution of diameters of each nanofibrous mat. PVA is easily electrospun and yields uniform nanofibers with smaller diameter. However, crosslinked PVA exhibited a slight higher diameter with an average of approximately 120 nm. It was observed that PVA/CMC-1 nanofibers also presented a uniform diameter range starting from 80 to the maximum diameter of approximately 180 nm with an average diameter of 120 nm, which was also an indication towards successful nanofibers production using the stated ratios of PVA and MC. Inversely, no nanofiber was observed in case of PVA/CMC-2 (having PVA:CMC = 6:2) rather it was rather electrosprayed. PVA/PVP nanofibers were also uniform however these nanofibers had a larger diameter than that of pure PVA and PVA/CMC nanofibers. Tricomponent nanofibers C1P12, C2P12, and C3P12 also exhibited uniform diameters ranging from 50 to 400 nm. Slight diversity was observed due to variation in viscosity and conductivity of solution caused by varying concentration of CMC. It was concluded that samples having 12% PVP and varying concentration of CMC (1–3 weight %) exhibited uniform nanofibers without beads formation, and uniform diameter distribution.

### 4.3. Air Permeability

Nanofiber based food packaging have an advantage over paper based or film based food packaging because of the unique porous structure of nanofiber mats. Air permeability (AP) allows food items especially fruits and vegetables to breathe (proper inhale/exhale of oxygen and carbon dioxide), which keeps them fresh for a longer period of time. Table 2 represents air permeability of PVA, PVA/PVP, PVA/CMC, and PVA/PVP/CMC nanofiber mats. It can be observed that all samples exhibited excellent air permeability. AP values for PVA/PVP were recorded higher than other samples, which may be because of higher porosity and lower fiber density of PVA/PVP. However, PVA/PVP/CMC nanofibers also exhibited better AP, which can be considered as feasible values for food packaging applications. Air permeability never means that bacteria, viruses, or other pathogens can also pass through pores among nanofibers. Pore size of nanofiber mats is usually larger than the particle size of air and smaller than that of bacteria, viruses, and other harmful pathogens [48]. That is one of the reasons that nanofiber based food packaging will be superior to ordinary food packaging materials.

### 4.4. Water Contact Angle (WCA)

**Figure 4:**
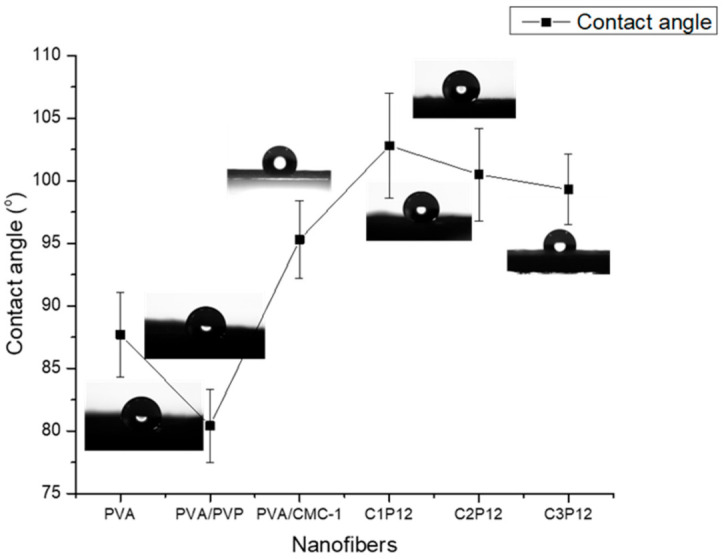
Water contact angles (WCA) of PVA, PVA/PVP, and CMC based composite nanofibers.

The hydrophilic or hydrophobic nature of polymeric nanofibers plays an important role while targeting final applications [27]. Food packaging should be hydrophobic to sustain in the wet and moist environment. Hydrophilicity of substance may be analyzed by calculating the water contact angle (WCA). WCAs of crosslinked PVA, PVA/PVP, and CMC based composite nanofibers were calculated to evaluate the degree of hydrophilicity/hydrophobicity of nanofibrous mats. It was stated in the literature that nanofibers of uncrosslinked PVA tend to be hydrophilic while the water contact angle increases with an increasing degree of crosslinking. It also depends on the type of crosslinking agents used to crosslink PVA, and the method of crosslinking as well [46]. Figure 4 shows contact angles for each sample. It can be observed that the water contact angle of crosslinked PVA was observed at 87.7° while it shows a slight decrease (80.4˚) in the case of PVA/PVP nanofibers. The addition of CMC caused an increase in the water contact angle of nanofiber mats. WCA for PVA/CMC, C1P12, C2P12, and C3P12 were recorded at 95.3°, 102.8°, 100.5°, and 99.3° respectively. An increase in the contact angle may be because of interchain and intrachain bonding of PVA, CMC, and PVP in the result of crosslinking. It was concluded that all nanofibers containing CMC possess enough hydrophobicity to be used in food packaging applications. However, hydrophobicity may be increased by further crosslinking of nanofibers but that will lead to the reduction of biodegradability of CMC based nanofibers.

### 4.5. Thermogravimetric Analysis (TGA)

**Figure 5:**
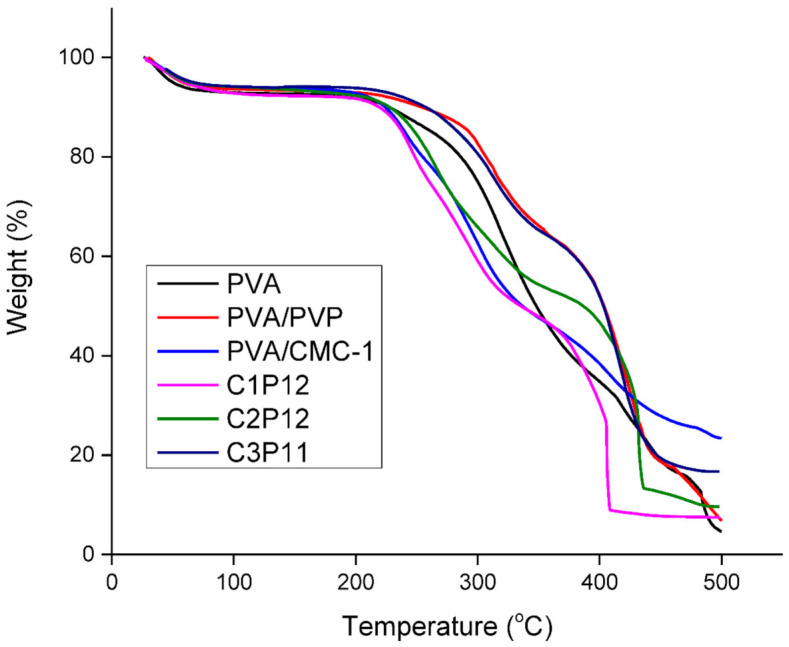
Thermogravimetric analyzer (TGA) plots of PVA, PVA/PVP, and CMC based composite nanofibers representing thermal degradation behavior of nanofibers.

PVA and PVP show thermal stability above 300 ℃ while CMC is thermally unstable above 280 °C. Figure 5 shows TGA curves of PVA/PVP and CMC based composite nanofibers in which ratios of PVP and PVA is constant and concentration of CMC changes. Generally there are three parts of TGA plot depending on temperature zones [49]. The first zone indicates the evaporation of impurities, vapors and high volatile components and this zone is up to 100 °C. The second zone shows the thermal stability of the material. It lies between onset temperatures and offset temperature. The last and third temperature zone starts from the offset temperature, which indicates the degradation of material. In the third zone, thermally unstable materials show a flatter line than the materials that are thermally stable. It can be seen that PVA/PVP nanofibrous mats show thermal stability well above 300 °C while onset temperature was dropped to 220–250 °C for nanofibers containing CMC, which shows that addition of CMC in tricomponent nanofibrous mats caused a decrease in thermal stability of polymers. However, thermal stability up to 200 °C is good enough for food packaging application.

### 4.6. X-ray Diffraction (XRD)

**Figure 6:**
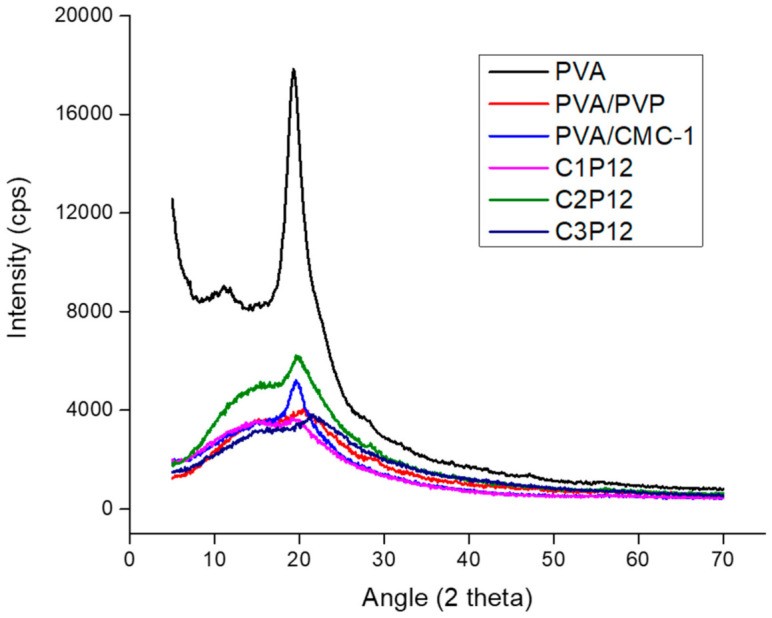
X-ray diffraction (XRD) spectra of PVA, PVP, and PVA/PVP/CMC nanofibers.

Crystalline structure of PVA nanofibers was compared with that of tricomponent composite nanofibers of PVA/PVP/CMC by the XRD spectra. It can be observed (Figure 6) that PVA nanofibers exhibited the characteristic peak amorphous structure of PVA at 2θ = 19.6°. However the characteristic peak of PVA was suppressed by the addition of PVP, and a further shift was observed in the case of CMC addition. CMC generally exhibits its characteristic peak at 22° [50] while PVP exhibits its characteristic peak at 21.84° [51]. It was observed that addition of CMC caused a slight shift in peak towards 21–22°, which shows the dominance of CMC is tricomponent composite nanofibers. However, PVP had also a peak at 21.84° [52], it could also be an effect of PVP but the intensity of tricomponent peaks tended towards CMC as shown in Figure 6. It was further evaluated for mechanical properties to conform to XRD results and it was observed that mechanical properties of nanofiber mats were also affected by the addition of CMC and PVP.

### 4.7. Mechanical Properties

Mechanical properties are important for any material, which is designed for commercial applications such as packaging. Tensile strength was measured for PVA, PVA/PVP, PVA/CMC, and tricomponent PVA/PVP/CMC nanofibers, and the effect of loading of CMC on the mechanical properties of the final product was compared with that of pure PVA or PVA/PVP nanofibers. Table 3 represents the mechanical properties of PVA, PVA/PVP, PVA/CMC, and tricomponent PVA/PVP/CMC nanofibers. It can be shown that PVA/PVP nanofibers had significant tensile strength, however strain (elongation at break) was increased, which indicates that PVA/PVP nanofibers were more elastic than plastic. However, trends were observed to be opposite in the case of CMC. PVA/CMC nanofibers exhibited higher tensile strength (as compared to PVA/PVP nanofibers) and lower tensile strain, which indicates the presence of a crystalline lattice in the main structure of CMC as that of PVP. It can be observed that all samples possess good tensile strength, however tensile strength of PVA/PVP was dropped to 8.2 MPa. All other nanofiber mats exhibited a tensile strength over 10 MPa, which is considered enough in the case of nanofibers. It was also concluded that these nanofiber mats have enough mechanical strength to be used for food packaging applications.

### 4.8. Compatibility Study (Food Packaging)

**Figure 7:**
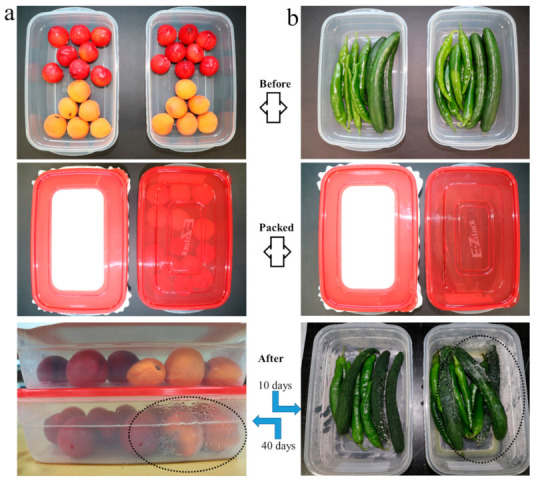
Real time observatory experiments for suitability of nanofibers based food packaging (**a**) for fruits up to 40 days and (**b**) for vegetables up to 10 days; relative humidity was 75% at 2 °C temperature (containers with red translucent lids are plastic based, while white lids represent nanofibers based lids.)

An experimental study was carried out to investigate suitability potential of nanofibers based food packaging by comparing with plastic based packaging. Fruits and vegetables of a similar type and freshness were taken and packed in different containers. One container for each type was packed by the plastic lid and the other was packed by nanofiber mats (C1P13 samples were used in this experiment, nanofiber based containers have white fibrous mats fixed in red bordered lids). All samples were placed in a controlled environment in the same refrigerator. Vegetables were examined after 10 days while fruits were placed for 40 days. Figure 7 shows the results obtained by the experiment. It can be observed that fruits and vegetables packed in nanofiber based packaging were having better freshness than that packed by the plastic one. There may be several reasons behind these results and two of those are; firstly nanofibers that have excellent air permeability and secondly nanofiber mats possess excellent breathability (moisture vapor transport rate) as compared to that of the plastic sheet/film. The presence of water vapors in plastic based food packaging is circled in Figure 7, which supports our claim. It can be concluded that nanofibers based food packaging is superior to conventional food packaging, and it can significantly enhance shelf life of fruits and vegetables.

## 5. Conclusions

CMC was selected as a target material for electrospun nanofiber based food packaging due to biocompatibility, biodegradability, and sustainability. From the results of morphological characterization, it was concluded that CMC could be electrospun along with PVA and PVP, and possess uniform morphology. Diameters were also found to be in uniform range. The water contact angle was also measured to confirm the stability of nanofibrous mats in outdoor use. It was concluded that all nanofibers were found to be hydrophobic. It was because of crosslinking of PVA, PVP, and CMC. In general all these polymers are hydrophilic and water soluble in the uncrosslinked form, however, these were transformed to being water insoluble and hydrophobic due to crosslinking. Tensile properties were also found to be conformed to the target application as all nanofibers of the tricomponent blend had tensile strengths well above 10 MPa. Air permeability is one of the important factors that were evaluated for nanofibrous mats and it was concluded that all nanofibrous mats possess excellent air permeability, which will keep fruits and vegetable fresh for a longer time. It was also observed in the results that fruits and vegetables closed by nanofibers based webs exhibited better freshness and lower accumulation of moisture in the containers. Considering all results it is suggested that PVA/PVP/CMC nanofibrous mats can be used for food packaging.

## Figures and Tables

**Table 1:** Polymer composition in tri-component composite nanofibers.

Sample Names	Polymers’ Composition (Weight %)			Observations
	PVA	PVP	CMC	
PVA/CMC-1	6	0	1	Fibers observed
PVA/CMC-2	6	0	2	Electrospraying (No nanofiber)
PVA/PVP	6	12	0	Fibers observed
C1P12	6	12	1	Fibers observed
C2P12	6	12	2	Fibers observed
C3P12	6	12	3	Fibers observed

**Table 2:** Air permeability of PVA, PVA/PVP, and CMC based composite nanofibers.

Samples	Air Permeability (Liter/Minute)
PVA	37.01 ± 2.04
PVA/PVP	52.66 ± 2.98
PVA/CMC-1	36.18 ± 1.97
C1P12	42.75 ± 2.45
C2P12	41.03 ± 2.61
C3P12	39.81 ± 1.87

**Table 3:** Tensile strength and elongation at break of PVA, PVA/PVP, PVA/CMC, and tricomponent nanofibers.

Samples	Tensile Strength (MPa)	Elongation at Break (%)
PVA	11.57 ± 0.56	22.27 ± 3.14
PVA/PVP	8.61 ± 0.49	31.36 ± 2.94
PVA/CMC-1	11.37 ± 0.48	18.91 ± 2.47
C1P12	10.29 ± 0.37	29.91 ± 3.89
C2P12	10.33 ± 0.6	25.91 ± 2.97
C3P12	11.05 ± 0.58	20.38 ± 3.09
